# Variability in variability: does variation in morphological and physiological traits differ between men and women?

**DOI:** 10.1098/rsos.230713

**Published:** 2023-09-06

**Authors:** Lewis G. Halsey, Gabriel P. Esteves, Eimear Dolan

**Affiliations:** ^1^ School of Life and Health Sciences, University of Roehampton, London SW15 4JD, UK; ^2^ Applied Physiology and Nutrition Research Group, Center of Lifestyle Medicine, Faculdade de Medicina FMUSP, Universidade de São Paulo, São Paulo, SP, Brazil

**Keywords:** greater male variability, variation, human traits, sexual dimorphism, menopause, menstrual cycle

## Abstract

Many researchers presume greater variability between female participants than between males due to the menstrual cycle. This view has encouraged a sex bias in health and medical research, resulting in considerable knowledge gaps with important clinical implications. Yet in another field—evolutionary biology—the received wisdom is the reverse: that men are more variable, possibly due to male heterogamety. To test these competing hypotheses, we compared variance between the sexes for 50 morphological and physiological traits, analysing data from the NHANES database. Nearly half the traits did not exhibit sexual dimorphism in variation, while 18 exhibited greater female variation (GFV), indicating GFV does not dominate human characteristics. Only eight traits exhibited greater male variation (GMV), indicating GMV also does not dominate, and in turn offering scant support for the heterogamety hypothesis. When our analysis was filtered to include only women with regular menstrual cycles (and men of equivalent age), the number of traits with GFV and GMV were low and not statistically different, suggesting that the menstrual cycle does not typically explain GFV when it occurs. In practical terms, health and medical researchers should no longer simply assume that female participants will induce additional variation in the traits of interest.

## 1. Introduction

Human beings vary from one another in every characteristic imaginable—an obvious yet intriguing aspect of the human condition. The earliest statistical analyses of human variation were conducted by Karl Pearson on measurements of various body parts, where for the first time he deployed his now famous Pearson coefficient of variation [1]. In his report, he tentatively concluded ‘a slightly greater female variability’, i.e. that if anything body shape varies slightly more between women than between men. Shortly afterwards, Leta Hollingworth garnered morphological measurements for 1000 male and 1000 female new-borns in New York; taking advantage of Pearson's statistical approach, she observed some traits varying more in little boys, some traits varying more in little girls, and other traits showing no sexual dimorphism in variability [2; their table N].

Within many fields today, including medical and physiological, it is typically assumed that inter-individual variation between females is greater than between males due to the hormonal fluctuations that occur across the menstrual cycle [3–5]. This supposed additional variation is believed to complicate research designs and decrease statistical power [6], and is commonly deployed as justification for excluding women from health and medical studies. By contrast, men are asserted to be more physiologically consistent over time—a simpler model that induces less irksome variability into a study. Consequently, the literature is based predominantly on measurements taken from men [7–9]. This disparity has created crucial knowledge gaps even within extensively researched clinical areas [10]. For example, there are well-documented misdiagnoses and inadequate treatment prescriptions for women [11–13], and many approved drugs have subsequently been found to have more adverse effects on women [10,14–16].

Many researchers within the health and medical fields will be unaware that an opposing hypothesis related to sex differences in variability exists within the field of evolutionary biology—a hypothesis that has been around since the time of Darwin and Wallace, and purports that in fact it is males who display greater variability. And, greater male variability (GMV) in humans has been reported a number of times using modern analyses of relatively large sample sizes, including for birth weight, adult weight and height, body mass index and brain structure [17–20]. Many cognitive traits also demonstrate GMV, including creativity [21,22], general knowledge [23], time, risk and social preferences [24] and various intellectual faculties [25–33] (cf. [23]). Several explanations for GMV have been proposed and explored. One is that the stronger sexual selection experienced by males results in sexual traits exhibiting greater inter-individual variance among males than among females [34,35]; see [36] for a detailed explanation. Another is heterogamety—the occurrence of homogametic sex chromosomes in females and heterogametic sex chromosomes in males [37], resulting in the expectation that recessive X-chromosome genes will be expressed in males more frequently or strongly than in females [38].

However, there is evidence that conflicts with the claim in some fields of the predominance of GMV. Of 31 blood parameters, Lehre *et al*. [17] observed that 13 exhibit GMV while seven exhibit greater female variation (GFV), and 11 exhibit neither GMV nor GFV. In mice, Zajitschek *et al*. [39] tested the variation of a gamut of diverse traits and in summary found that while morphological traits typically exhibit GMV, no other trait categories do, while immunological traits and eye function traits generally exhibit GFV. They suggest the aforementioned mechanisms as possible explanations for this GMV and GFV.

There is, then, far from a consensus on whether it is men or women who display more inter-individual variability, and under what circumstances. And this is not just a question of biological interest but also has practical relevance. There are clinical implications of the putative presence of GMV or GFV because appropriate diagnosis and treatment not only depend on understanding the magnitude of relevant trait differences between the sexes but also the extent of variation in those traits between individuals. Understanding inter-individual variability in men compared to women requires large, robust sample sizes for a diversity of trait types. The National Health and Nutrition Examination Survey (NHANES) is an ideal resource for this objective. This survey is described as ‘a program of studies designed to assess the health and nutritional status of adults and children in the United States' (https://www.cdc.gov/nchs/nhanes/about_nhanes.htm). Each year, through this survey, NHANES records a plethora of health and nutrition data for citizens around the USA, with all measurements being archived and made freely available online. We took advantage of this extensive database to study and compare the degree of variation between men and women in characteristics including body morphometrics, blood counts, basic cardiovascular function and biochemistry profiles. This enabled us to assess the degree of evidence, based on the NHANES data, for the competing hypotheses that GMV predominates (due to heterogamety or stronger sexual selection in males) versus that GFV predominates (due to hormonal and physiological effects of the menstrual cycle) in human non-cognitive traits. This study is the most extensive investigation to date into the nature and prevalence of human sexual dimorphism in variation of non-cognitive traits, in terms of the number of traits considered and the sample sizes assessed.

## 2. Methods

The NHANES survey recruits participants through invitation only, based on random selection by applying a statistical process that uses USA census information. We downloaded and combined NHANES data for 2009–2020, for variables hereby categorized into functional groups following the NHANES nomenclature as: body morphometrics, cardiovascular function, blood counts, biochemistry profiles and daily energy intake. These variables can be accessed through their corresponding NHANES data subsections, i.e. *‘*Examination Data’ for body morphometrics and cardiovascular function; ‘Laboratory Data’ for blood counts and biochemistry profiles; and ‘Dietary Data’ for daily energy intake. Our aim was to explore a range of traits, to identify whether GMV or GFV broadly dominate. To achieve this, we randomly selected from those traits available that were (a) quantitative rather than qualitative and (b) had been collected for many years with a consistent method according to the NHANES accompanying information. Fifty variables were selected to ensure that this initial analysis of the NHANES dataset for sexual dimorphism in inter-individual trait variability was both robust and manageable. We derived two variables from downloaded variables: mean total leg mass was calculated as the average of left and right total leg masses, while daily energy intake was calculated as the average of the two one-day intake values that were available. We also downloaded demographic data on age and sex. Once the data for children (less than 18 years old) were removed, the dataset comprised 33 338 individuals (16 163 men and 17 175 women). The distribution of ages for the two sexes was very similar (men: mean 49.2 ± s.d. 18.3; women: 49.0 ± 18.1). A small percentage of data points (always less than 2%) were missing for each of the variables within the years that those data were reported. Sample sizes per variable are provided in the results. We undertook all analyses of these data in R v. 3.5.3.

Weighting must be applied to account for the complex survey design used by NHANES along with survey non-response, differential probabilities of selection for the sampling domains, post-stratification adjustment and any differences between the final sample distribution from the target population distribution. In our analysis, we used the sample weights calculated and provided by NHANES, adjusted to accommodate our multi-cycle analysis. These sample weights were then applied using the survey() package [40]. This weighting is a measure of the number of people in the population represented by that sample person, having accounted for the oversampling of some demographics and non-responses, so that the produced estimates and analysis from the data are representative of the civilian, non-institutionalized US population. We then disaggregated the data into men and women. Coefficients of variance were calculated for each variable, per sex. The natural log of the ratio of the male to female coefficients of variance was computed to provide an unbiased ratio of GMV or GFV in the sample—natural log ratios greater than 0 indicated GMV within the sample and values less than 0 indicated GFV. To determine whether the degree of GMV or GFV observed in the sample is statistically significant, the difference in the coefficient of variance for males and for females was calculated and the boot() package [41] was then used to generate associated 95% confidence intervals around that difference. The bootstrap method generates confidence intervals around estimates by using a resampling with replacement technique, in which *n* observations are drawn *i* times (*i* = 1000 in this case) and the statistic of interest (coefficient of variance) is calculated every iteration. These multiple calculated statistics form a new distribution, from which the 95% confidence interval is derived by selecting the 2.5th and 97.5th percentiles [42]. When those confidence intervals do not encompass 0, the associated *p*-value < 0.05.

We then reran the analyses to investigate if and how the results differed when women were represented by only those self-identifying as having regular periods (and thus not perimenopausal, menopausal or post-menopausal). This was achieved by sub-setting the dataset by answers to the NHANES reproductive health question entitled ‘had regular periods in past 12 months'. After this disaggregation, all men and remaining women more than 50 years of age were removed from the dataset to minimize a potentially confounding influence of age. We then equalized the sample sizes of the men and women by randomly sub-sampling the men to a number equal to that of the (smaller) number of women (*n* = 4118 in each group). The distribution of ages for the two sexes was very similar (men: mean 33.6 ± s.d. 9.1; women: 33.1 ± 8.8). Although the sample size was still large, it was considerably reduced from the sample size for the entire dataset. To account for this, specifically for comparison against the subset of data including only women who were having regular periods, a dataset of equivalent sample size was generated by randomly sub-sampling the full dataset.

Significant differences in the number of traits presenting with GMV versus GFV were tested for with χ^2^ goodness-of-fit tests, where expected values were set as 50% of the total number of traits exhibiting GMV or GFV.

## 3. Results

**Figure 1.**
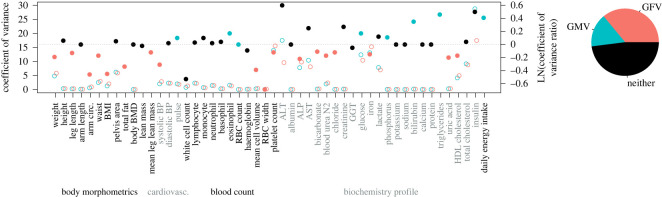
The magnitudes of variation within all men (open green-blue circles) and within all women (open red circles) in the dataset for morphometric and physiological traits, and also daily energy intake, are scaled against the left-hand *y*-axis. The natural log values of the ratio of those variations for males and females are scaled against the right-hand *y*-axis (filled circles: green-blue, greater male variation; red, greater female variation; black, neither). The left *y*-axis is truncated at 30 so that differences in the coefficient of variance between males and females can be clearly viewed for most traits; however, one or both values for total fat, lean mass, mean leg lean mass, RBC width, GGT, triglycerides and daily energy intake is not plotted in the figure. Note that visually indiscernible differences in the variation of a trait between men and women nonetheless can sometimes be accompanied by a statistically significant greater male or female variation due to real differences in the degree of variation between the sexes coupled with relatively small 95% confidence intervals, an example of this being mean cell volume. The pie chart summarizes the proportions of traits presenting with GMV, GFV and neither. See table 1 for acronym definitions.

Of the 50 traits we analysed, for the full dataset of 33 338 people (i.e. including men and women of all observed ages), eight statistically significantly presented with GMV, 18 with GFV and 24 with neither (table 1 and figure 1). Thus neither GMV nor GFV predominate in these traits; however, there is a statistically significantly higher frequency of GFV than GMV (*χ*^2^ = 3.85, *p* = 0.050). GFV was also present at a higher frequency than GMV in the body morphometrics trait group—there was GFV in seven traits while GMV was not present in any of them (*χ*^2^ = 7.00, *p* = 0.008). There were no differences in the frequencies of GFV and GMV for either of the trait groups blood count (2 versus 2; *χ*^2^ = 0, *p* = 1) and biochemistry profile (7 versus 4; *χ*^2^ = 0.818, *p* = 0.366); only three traits represent the group cardiovascular function so this group was not statistically analysed.

**Table 1.** The means and coefficients of variances of 50 morphological and physiological traits, and daily energy expenditure, within men and within women. Also presented are the ratios and log ratios of those variances, the differences between those variances, the 95% confidence intervals around those differences and the interpretation of those intervals. Sample sizes per trait are also provided. These data are visualized in figure 1. BMI, body mass index; BMD, body mineral density; BP, blood pressure; RBC, red blood cell; ALT, alanine aminotransferase; ALP, alkaline phosphatase; AST, aspartate aminotransferase; GGT, γ-glutamyl transferase; HDL, high density lipid; GFV, greater female variance; GMV, greater male variance.

	male mean	female mean	male *N*	female *N*	male CV	female CV	CV ratio	ln (CV ratio)	CV difference	lower 95% CI	upper 95% CI	sexual dimorphism?
*body morphometrics*												
weight (kg)	89.3	76.72	15 189	16 178	4.81	5.79	0.83	−0.19	−0.98	−1.24	−0.72	GFV
height (cm)	175.56	161.63	15 187	16 175	0.33	0.31	1.06	0.06	0.01	0	0.02	
leg length (cm)	41.21	37.08	14 561	15 250	0.23	0.26	0.88	−0.13	−0.03	−0.04	−0.02	GFV
arm length (cm)	39.15	35.9	14 716	15 501	0.14	0.14	1	0	0	0	0	
arm circumference (cm)	34.39	32.22	14 710	15 502	0.59	0.94	0.63	−0.46	−0.35	−0.38	−0.32	GFV
waist (cm)	101.61	97.19	14 600	15 274	2.49	2.95	0.84	−0.17	−0.46	−0.56	−0.36	GFV
BMI (kg m^−2^)	28.9	29.34	15 153	16 155	1.29	2.02	0.64	−0.45	−0.73	−0.81	−0.65	GFV
pelvis area (cm^2^)	220.13	185.35	4871	4620	6.2	5.9	1.05	0.05	0.3	−0.08	0.68	
total fat (g)	286 72.29	264 44.76	4628	4602	1448.15	2034.66	0.71	−0.34	−586.51	−738.44	−428.35	GFV
body BMD (g cm^−^^2^)	1.14	1.08	4530	4471	0.01	0.01	1	0	0	0	0	
lean mass (g)	609 65.91	442 22.47	4551	4551	1715.25	1753.07	0.98	−0.02	−37.82	−163.92	86.48	
mean leg lean mass (g)	247 51.42	306 69.54	4517	4456	4336.22	4845.91	0.89	−0.12	−509.69	−906.03	−127.47	GFV
*cardiovascular function*												
systolic BP (mmHg)	123.42	119.61	14 472	15 071	2.04	2.8	0.73	−0.31	−0.76	−0.88	−0.64	GFV
diastolic BP (mmHg)	71.9	69.18	14 472	15 071	2.28	2.23	1.02	0.02	0.06	−0.08	0.21	
pulse (beats min^−1^)	70.76	73.75	14 316	14 960	2.03	1.83	1.11	0.1	0.21	0.13	0.28	GMV
*blood count*												
white cell count (1000 cells µl^−1^)	7.19	7.33	14 619	15 603	0.77	1.31	0.59	−0.53	−0.54	−3.25	2.18	
lymphocyte (%)	29.63	30.53	14 610	15 577	2.28	2.21	1.03	0.03	0.07	−0.03	0.17	
monocyte (%)	8.48	7.59	14 610	15 577	0.69	0.62	1.11	0.1	0.06	−0.02	0.14	
neutrophil (%)	58.21	58.63	14 610	15 577	1.45	1.42	1.02	0.02	0.03	−0.03	0.09	
basophil (%)	0.75	0.75	14 610	15 577	0.27	0.26	1.04	0.04	0.01	−0.02	0.04	
eosinophil (%)	3.01	2.57	14 610	15 577	1.53	1.3	1.18	0.17	0.23	0.09	0.37	GMV
RBC count (million cells µl^−1^)	4.92	4.45	14 619	15 603	0.04	0.04	1	0	0.01	0.01	0.01	GMV
haemoglobin (g dl^−1^)	15.04	13.35	14 619	15 603	0.1	0.11	0.91	−0.09	−0.01	−0.02	0	
mean cell volume (fl)	89.9	89.12	14 619	15 603	0.27	0.4	0.68	−0.39	−0.13	−0.15	−0.11	GFV
RBC width (%)	13.2	13.48	14 619	15 603	0.08	0.16	0.5	−0.69	−0.07	−0.08	−0.06	GFV
platelet count (1000 cells µl^−1^)	225.8	252.35	14 619	15 602	13.93	15.57	0.89	−0.12	−1.64	−2.49	−0.79	GFV
*biochemistry profile*												
ALT (U l^−1^)	29.26	20.76	14 381	15 263	17.51	9.61	1.82	0.6	7.9	−1.25	17.15	
albumin (g dl^−1^)	4.36	4.17	14 384	15 270	0.03	0.03	1	0	0	0	0	
ALP (IU l^−1^)	68.35	68.67	14 380	15 267	7.8	9.79	0.8	−0.22	−1.99	−3.91	−0.01	GFV
AST (U l^−1^)	26.93	23.11	14 358	15 248	10.45	8.12	1.29	0.25	2.33	−4.09	8.86	
bicarbonate (mmol l^−1^)	25.45	24.87	14 383	15 267	0.19	0.21	0.9	−0.11	−0.02	−0.03	−0.01	GFV
blood urea N_2_ (mg dl^−1^)	14.63	13.11	14 384	15 265	1.99	2.36	0.84	−0.17	−0.37	−0.62	−0.13	GFV
chloride (mmol l^−1^)	102.99	103.47	14 383	15 270	0.08	0.09	0.89	−0.12	−0.01	−0.01	−0.01	GFV
creatinine (mg dl^−1^)	1	0.78	14 384	15 268	0.17	0.13	1.31	0.27	0.04	−0.05	0.13	
GGT (U l^−1^)	31.77	23.34	14 381	15 266	55.07	57.8	0.95	−0.05	−2.73	−33.54	28.05	
glucose (mg dl^−1^)	101.99	97.83	14 384	15 268	12.4	10.45	1.19	0.17	1.95	0.14	3.81	GMV
iron (μg dl^−1^)	94.16	79.52	14 366	15 252	13.14	15.23	0.86	−0.15	−2.1	−2.87	−1.29	GFV
lactate (U l^−1^)	133.45	133.61	14 256	15 204	7.87	6.99	1.13	0.12	0.88	−1.71	3.57	
phosphorus (mg dl^−1^)	3.64	3.8	14 381	15 265	0.09	0.08	1.12	0.11	0.01	0.01	0.01	GMV
potassium (mmol l^−1^)	4.06	3.95	14 379	15 264	0.03	0.03	1	0	0	0	0	
sodium (mmol l^−1^)	139.55	139.3	14 383	15 270	0.04	0.04	1	0	0	0	0	
bilirubin (mg dl^−1^)	0.71	0.55	14 377	15 258	0.17	0.12	1.42	0.35	0.04	0.02	0.05	GMV
calcium (mg dl^−1^)	9.42	9.36	14 364	15 256	0.01	0.01	1	0	0	0	0	
protein (g dl^−1^)	7.14	7.05	14 356	15 262	0.03	0.03	1	0	0	0	0	
triglycerides (mg dl^−1^)	166.57	133.75	14 379	15 256	126.57	79.73	1.59	0.46	46.83	9.1	86.37	GMV
uric acid (mg dl^−1^)	6.04	4.79	14 382	15 262	0.27	0.33	0.82	−0.2	−0.07	−0.08	−0.06	GFV
HDL cholesterol (mg dl^−1^)	48.12	58.94	14 428	15 326	4.14	4.93	0.84	−0.17	−0.79	−1.03	−0.55	GFV
total cholesterol (mg dl^−1^)	188.16	194.93	14 428	15 326	9.18	8.8	1.04	0.04	0.39	−0.07	0.86	
insulin (μU ml^−1^)	14.41	12.97	6901	7381	28.77	17.46	1.65	0.5	11.31	−2.32	25.35	
daily energy intake (kcal)	2435.22	1786.56	14 144	14 934	339.16	224.42	1.51	0.41	114.74	97.24	132.5	GMV

The randomly selected subset presented five traits with GMV, 14 with GFV and 31 with neither, again representing a statistically significantly higher frequency of GFV than GMV (*χ*^2^ = 4.26, *p* = 0.039), whereas the reduced dataset restricted to under 50-year-olds and women with a regular menstrual cycle presented 10 traits with GMV, 14 with GFV and 26 with neither, representing a statistically non-significant difference in frequencies of GFV and GMV (*χ*^2^ = 0.67, *p* = 0.414) (electronic supplementary material, figure S1).

## 4. Discussion

Evolutionary biologists have debated the prevalence of GMV for well over a century [36,43–45], arguing it is caused by heterogamety or high genetic variance in male sexually selected traits. In stark contrast, the medical and physiological literature leans towards a received wisdom of GFV, typically based on the suggestion that the menstrual cycle induces additional variability in labile traits. Which is true? Is it the case that human characteristics predominantly exhibit with GMV, or with GFV, or neither?

GFV is present in 18 of the traits we analysed—a minority of them. GMV is present in fewer—only eight of the traits (figure 1 and table 1). Moreover, GMV is not present in any morphological characteristics, which researchers often report as showing greater variation in males [17,37,39]. So both GMV and GFV are exhibited in only a minority of all the traits measured. Indeed, nearly half of characteristics do not differ in degree of inter-individual variability between the sexes. Therefore, our analysis of over 30 000 adult individuals has put to rest the idea that either GMV or GFV is anywhere near ubiquitous, at least within the functional groups of traits that we investigated.

However, the greater presence of GFV than GMV is statistically significant. Could this be explained by the hypothesis that hormonal fluctuations throughout the menstrual cycle increase female variability and thus inter-individual variability? We re-ran the dataset with women represented only by those who were experiencing regular menstrual cycles and thus subject to the hormonal fluctuations that characterize a eumenorrheic cycle (electronic supplementary material, figure S1) and men of the same age, and compared this to a randomly selected subset of data of the same size from the full dataset. The randomly selected subset shows 14 traits presenting with GFV and five with GMV—both slightly lower absolute counts than present in the full dataset (explainable by the reduction in sample size). The subset represented by women with regular menstrual cycles again shows 14 traits presenting with GFV. Moreover, a number of the traits exhibiting GFV are morphometric (e.g. weight and arm circumference), considered not to be labile at least in the short term and thus not to have the potential to be affected by the menstrual cycle. Thus, there is no evidence from our analyses that GFV is driven by effects of the menstrual cycle.

With regards the albeit rather limited presence of GMV, we can test the hypothesis that it is exhibited in male sexually selected traits (due to high genetic variance), by investigating whether the traits considered most obviously to contribute to male reproductive success in the available dataset tend to be those (relatively few) that exhibit GMV. Lean muscle mass is a particularly interesting trait to consider in this regard, as greater muscle mass is purported to promote mating opportunities [46]. The traits associated with muscle (total lean mass, leg lean mass, and serum creatinine which positively associates with muscle mass) do not indicate GMV. Instead, the first two traits exhibit GFV, while serum creatinine does not exhibit sexual dimorphism in variation in either direction. Moreover, traits directly associated with body fat, which might be considered to underpin some female sexual characteristics—weight, waist circumference, BMI and total fat—all also exhibited GFV. In fact, over half of the morphological characteristics analysed presented with GFV—a surprising result that, as far as we are aware, has not previously been reported and is worthy of further investigation. Thus not only have we found the prevalence of GMV to be low but we find no suggestion that when it occurs it is driven by the riskier development of male sexual characteristics.

We note, though, that GMV is clearly present in daily energy intake, which reaffirms the strong GMV in daily energy expenditure reported in [47] given that energy intake usually matches energy expenditure over time [48,49]. As discussed in Halsey *et al*. [47], this GMV in energetics could be the result of GMV in a number of traits that drive metabolic rate, from organ size to activity levels.

## 5. Summary and conclusion

GFV driven by the menstrual cycle and GMV driven by heterogamety or sexual selection of male traits are diametrically opposed competing hypotheses about trait variability. Our data indicate that neither GFV nor GMV are anywhere close to dominating human characteristics, contra to some expectations in the fields of medicine and evolutionary biology, respectively. Indeed, nearly half of the traits we analysed exhibit no statistically significant difference in variability between men and women despite our very large sample sizes. And, while GFV was present in a number of traits, we found no evidence that this is in general due to the menstrual cycle. Research designs should no longer assume that women, or men, are likely to vary more in the characteristics to be measured, without direct prior evidence (the present study clearly provides this for some traits). This approach should further progress greater female participation in medical and clinical research. We hope that the exploratory study we report here catalyses further work into human variation and how it associates with sex and other factors in the pursuit of better understanding human diversity.

## Data Availability

The data are freely accessible on the NHANES website: https://www.cdc.gov/nchs/nhanes/index.htm.
